# Association between substance use and sleep disturbances among adolescents: Systematic review and meta-analysis

**DOI:** 10.1192/j.eurpsy.2025.656

**Published:** 2025-08-26

**Authors:** D. Phiri, V. L. Amelia, M. Muslih, L. P. Dlamini, M.-H. Chung, P.-C. Chang

**Affiliations:** 1 School of Nursing, College of Nursing, Taipei Medical University, Taipei, Taiwan, Province of China; 2 Nursing Department, Faculty of Health sciences, Universitas Muhammadiyah Purwokerto, Purwokerto; 3 School of Nursing, Faculty of Health Sciences, University of Muhammadiyah Malang, Malang, Indonesia; 4 Department of Nursing, Taipei Medical University Shuang Ho Hospital, Taipei, Taiwan, Province of China

## Abstract

**Introduction:**

Substance use is a major factor contributing to sleep disturbances in adolescents, whose developing brains are especially vulnerable. The previous studies have primarily focused on adults or individual substances.

**Objectives:**

This meta-analysis examined the overall association between substance use and sleep disturbances in adolescents.

**Methods:**

Relevant studies were systematically searched across multiple databases, including CINHAL (via EBSCOHOST), PubMed, Scopus, Ovid Medline, Embase, PsychINFO (via EBSCOHOST), and Web of Science, from inception until October 2021. A random-effects model was employed to calculate pooled Odds Ratios (OR) with 95% confidence intervals (CIs). Forest plots and Cochran’s Q statistic p values were utilized to assess heterogeneity among the studies. Subgroup and meta-regression analyses were conducted to compare groups and identify sources of heterogeneity. Study quality was evaluated using the Joanna Briggs Institute tool, and sensitivity analysis was performed to test the robustness of the results.

**Results:**

A comprehensive search identified 16,870 studies, of which 18 were included in the review and meta-analysis (Figure 1), published between 1993 and 2021. The studies were of high quality and had a low risk of bias. The results showed that substance use significantly contributes to sleep disturbances in adolescents (OR = 1.70, 95% CI: 1.49–1.94) (Figure2). Alcohol users, coffee drinkers, and smokers were significantly more likely to experience sleep disturbances (OR = 1.77, OR = 1.58; OR = 1.66), while marijuana showed a non-significant association (OR = 1.29) (Table 1). Additionally, alcohol and smoking were significantly associated with insomnia (OR = 1.82, 95% CI: 1.43- 2.33 and OR = 1.75, 95% CI: 1.31-2.33), hypersomnolence (alcohol: OR = 1.46, 95% CI: 1.18-1-81), and sleep-related breathing disorders (S-RBD) (alcohol: OR = 2.29, 95 % CI: 1.53-3.42; smoking: OR = 2.30, 95% CI: 1.23-4.30), with coffee also significantly associated with insomnia (OR = 1.58, 95% CI: 1.30-193). There was considerable heterogeneity among the studies, however, subgroup and meta-regression analysis indicated no statistically significant sources of heterogeneity.

**Table 1:** Association between individual substance use and sleep disturbances

	**n Study**	**OR**	**95% CI** **Lower limit**	**Upper limit**	**Q -value**	**Heterogeneity** **P-Value**	**I**^**2**^
Alcohol	15	1.77	1.55	2.03	102.21	<0.001	86.30
Coffee	3	1.58	1.30	1.93	6.02	<0.005	66.81
Marijuana	3	1.29	0.78	2.13	24.38	<0.001	91.79
Smoking	15	1.66	1.37	2.01	278.13	<0.001	94.96

*OR = Odds Ratio, CI = Confidence Interval*

**Image 1:**

**Figure:**
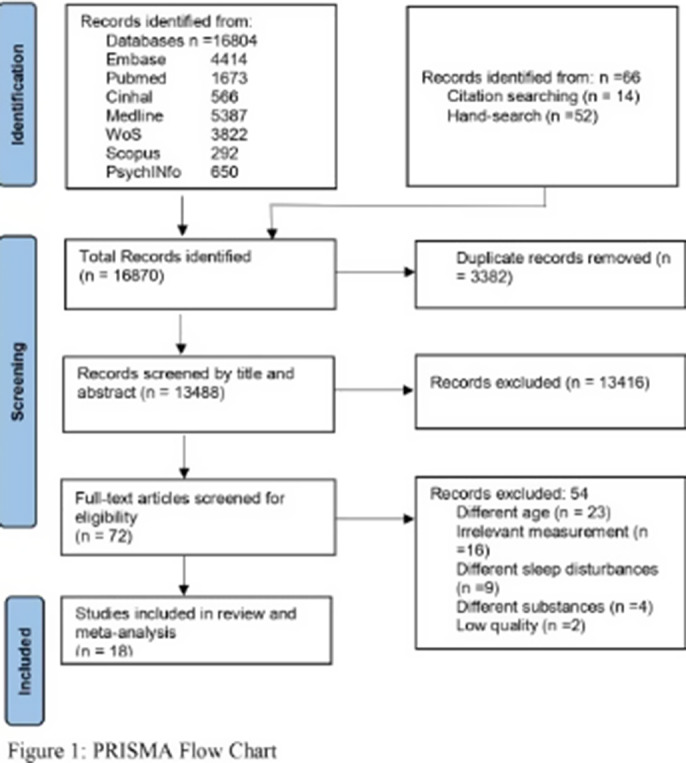

**Image 2:**

**Figure:**
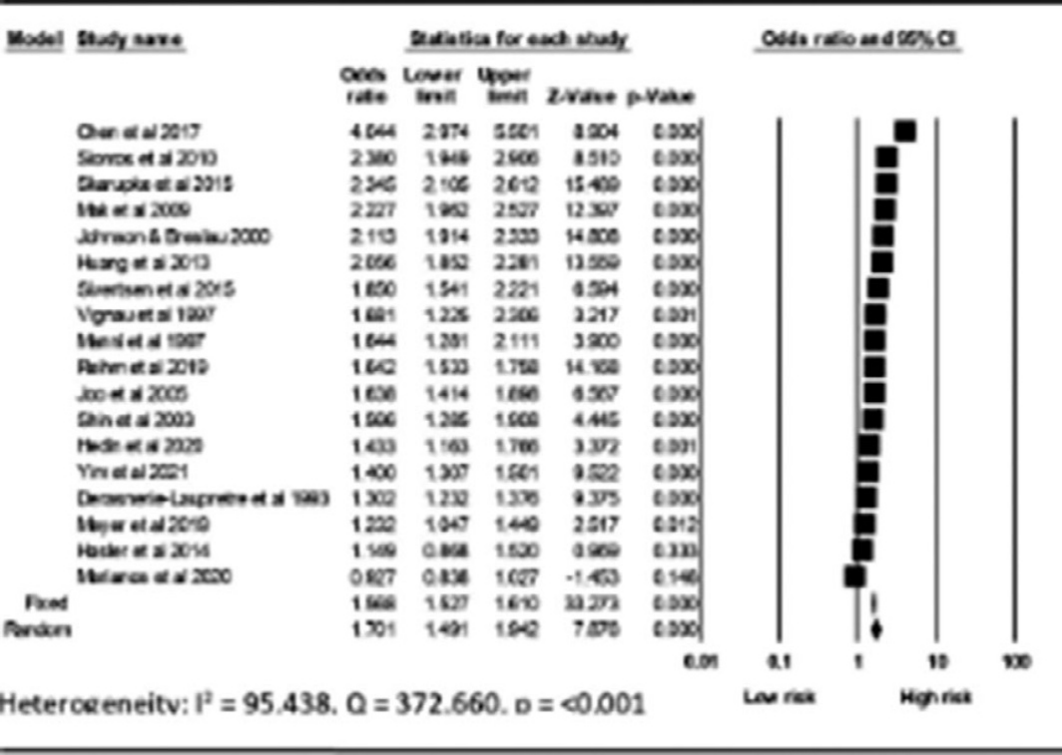

**Conclusions:**

Substance use significantly increases the risk of sleep disturbances in adolescents, with alcohol, coffee, and smoking showing strong associations. Despite the heterogeneity among studies, the findings underscore the need for targeted interventions to mitigate these risks and improve sleep health in this population.

**Disclosure of Interest:**

None Declared

